# Enhancing Caregiver Health: The Role of Psychoeducational and Exercise Interventions in Stress Management and Coping Among Dementia Family Caregivers

**DOI:** 10.1002/alz70858_106566

**Published:** 2025-12-26

**Authors:** Christopher H Herring, Brittany Butts, Melinda Higgins, Jordan Watson, Glenna S Brewster, Whitney Wharton, Rebecca Gary, Kenneth Hepburn, Sandra B Dunbar

**Affiliations:** ^1^ Emory University, Atlanta, GA, USA; ^2^ Emory University Nell Hodgson Woodruff School of Nursing, Atlanta, GA, USA; ^3^ Emory University, Nell Hodgson Woodruff School of Nursing, Atlanta, GA, USA

## Abstract

**Background:**

Black caregivers of persons living with Alzheimer's disease and related dementias (ADRD) experience significant health disparities, including elevated cardiovascular risk and high stress levels. This study evaluates the effects of a culturally tailored psychoeducational intervention, with and without exercise, on caregiver health, stress, and coping.

**Method:**

In this randomized controlled trial (NCT01188070), 142 Black caregivers were assigned to one of three groups: attention control, psychoeducation, or psychoeducation plus exercise. The psychoeducational intervention, *The Great Village*, focused on culturally relevant caregiving strategies, while the exercise component included a home‐based walking and resistance program. Outcomes assessed at baseline and six months included cardiovascular risk markers, perceived stress, and coping. Group differences were analyzed using ANCOVA, adjusting for baseline values.

**Result:**

The psychoeducation group showed significant reductions in diastolic blood pressure (*p* = 0.034) and blood glucose (*p* = 0.021). Both intervention groups reported lower perceived stress (*p* = 0.048). However, the psychoeducation plus exercise group did not demonstrate expected improvements in physical function or cardiovascular risk and showed a decline in positive reappraisal coping (*p* = 0.048).

**Conclusion:**

Culturally tailored psychoeducation may improve cardiovascular risk and stress outcomes in Black dementia caregivers. The added exercise component did not yield additional benefits, suggesting the need for alternative strategies to enhance physical activity in this population. Future research should explore longer‐duration interventions and ways to support exercise adherence within the context of caregiving.

